# Comorbidities Among Young Adults with Congenital Heart Defects: Results from the Congenital Heart Survey To Recognize Outcomes, Needs, and well-beinG — Arizona, Arkansas, and Metropolitan Atlanta, 2016**–**2019

**DOI:** 10.15585/mmwr.mm7006a3

**Published:** 2021-02-12

**Authors:** Matthew E. Oster, Aspen P. Riser, Jennifer G. Andrews, Elijah H. Bolin, Maureen K. Galindo, Wendy N. Nembhard, Charles E. Rose, Sherry L. Farr

**Affiliations:** ^1^National Center on Birth Defects and Developmental Disabilities, CDC; ^2^Department of Pediatrics, Emory University School of Medicine, Atlanta, Georgia; ^3^Children’s Healthcare of Atlanta, Atlanta, Georgia; ^4^Oak Ridge Institute for Science and Education, Oak Ridge, Tennessee; ^5^Department of Pediatrics, College of Medicine, University of Arizona, Tucson, Arizona; ^6^Section of Pediatric Cardiology, Department of Pediatrics, College of Medicine, University of Arkansas for Medical Sciences, Little Rock, Arkansas; ^7^Arkansas Center for Birth Defects Research and Prevention, Department of Epidemiology, Fay W. Boozman College of Public Health, University of Arkansas for Medical Sciences, Little Rock, Arkansas.

An estimated 1.4 million adults in the United States live with congenital heart defects (CHDs), yet their health outcomes are not well understood (*1*). Using self-reported, cross-sectional data from 1,482 respondents in the 2016–2019 Congenital Heart Survey To Recognize Outcomes, Needs, and well-beinG (CH STRONG) (*2*), CDC and academic partners estimated the prevalence of comorbidities among adults with CHDs aged 20–38 years born in Arizona (AZ), Arkansas (AR), and metropolitan Atlanta, Georgia (GA) compared with the general population (aged 20–38 years) from the National Health and Nutrition Examination Survey (NHANES) during 2015–2018 (*3*) and the AZ, AR, and GA Behavioral Risk Factor Surveillance Systems (BRFSS) during 2016–2018 (*4*). Adults with CHDs were more likely than those in the general population to report cardiovascular comorbidities, such as a history of congestive heart failure (4.3% versus 0.2%) and stroke (1.4% versus 0.3%), particularly those with severe CHDs (*2*). Adults with CHDs were more likely to report current depressive symptoms (15.1% versus 8.5%), but less likely to report previous diagnoses of depression (14.2% versus 22.6%), asthma (12.7% versus 16.9%), or rheumatologic disease (3.2% versus 8.0%). Prevalence of noncardiovascular comorbidities was similar between adults whose CHD was considered severe and those with nonsevere CHDs. Public health practitioners and clinicians can encourage young adults with CHDs to seek appropriate medical care to help them live as healthy a life as possible.

Adults with CHDs born during 1980–1997 were identified from population-based birth defects registries in AZ, AR, and GA. During October 2016–January 2019, eligible participants were surveyed regarding their CHDs, cardiovascular comorbidities, other comorbidities, quality of life, education, work history, and health care usage (*2*). Using questions from NHANES and BRFSS, CH STRONG participants were asked if they had ever been told by a doctor or other health professional that they had any cardiovascular comorbidities (congestive heart failure, hypertension, myocardial infarction, or stroke) or noncardiovascular comorbidities (asthma, cancer, mood disorder or depression, diabetes [type 1 or type 2, excluding gestational diabetes], or rheumatologic disease [arthritis, gout, lupus, or fibromyalgia]). Current depressive symptoms among adults with CHDs were assessed using the Patient Health Questionnaire-2 (PHQ-2) (*5*). Overweight/obesity was assessed using body mass index (BMI); persons with BMI ≥25 kg/m^2^ were considered to be overweight/have obesity. Among the general population, self-reported, clinician-diagnosed cardiovascular comorbidities and depressive symptoms (assessed by PHQ-2) were obtained from the 2015–2018 NHANES (*3*). Self-reported, clinician-diagnosed noncardiovascular comorbidities were obtained from the 2016–2018 BRFSS (*4*).

The CH STRONG sample was limited to persons aged 20–38 years (to match NHANES age groups for cardiovascular comorbidities) and to those without missing data for demographics or comorbidities. Furthermore, for the analyses of current depressive symptoms assessed using the PHQ-2, CH STRONG participants who did not self-report were excluded (to match the NHANES self-report data). To reduce nonresponse bias and generate population-based estimates, the CH STRONG participant population was standardized to the CH STRONG eligible population (9,312) by sex, birth cohort, maternal race/ethnicity, place of birth, and CHD severity. In addition, the general population samples (NHANES and BRFSS) aged 20–38 years were standardized to the CH STRONG eligible population by available demographic variables to reduce confounding. Standardized prevalence estimates were calculated for each cardiovascular and noncardiovascular comorbidity, as well as 95% confidence intervals (CIs) and p values for the difference in mean proportions between the standardized CH STRONG analytic sample and the standardized general population samples.

The CH STRONG sample was divided into two groups: those with severe CHDs and those with nonsevere CHDs (*2*). The unstandardized prevalence of cardiovascular and noncardiovascular comorbidities and the odds ratios between these two groups were measured and adjusted for sex, birth cohort, maternal race/ethnicity, and place of birth (adjusted odds ratios [aORs]). All analyses were conducted using SAS-callable SUDAAN (version 9.4; RTI International).

Among 9,312 eligible adults with CHDs, surveys were sent to 6,947 with available addresses; 1,656 surveys were returned (24% response rate). Of those, 1,626 were aged 20–38 years, 1,482 (91.1%) of whom included data on the variables of interest. Among these, 54% were female, 76% were non-Hispanic White, and the mean age was 26.1 (standard deviation = 4.6) years (Table 1). One third of the respondents had severe CHDs. A total of 1,174 eligible participants (79.2%) completed the survey via self-report, with the remainder by proxy.

**TABLE 1:** Selected characteristics of adults with congenital heart defects (CHDs) and the general population — United States, 2015–2019

Characteristic	CH STRONG 2016–2019*			General population			
				NHANES, 2015–2018^†^		BRFSS, 2016–2018^§^	
	Total (1,482)	Unstandardized, %	Standardized, %	Unstandardized weighted, %	Standardized weighted, %	Unstandardized weighted, %	Standardized weighted, %
**Sex**							
Female	805	54.3	48.8	49.7	48.6	48.0	48.6
Male	677	45.7	51.3	50.3	51.5	52.0	51.5
**Birth cohort** ^¶^							
1980–1985	214	14.4	14.5	35.4	14.4	25.9	14.4
1986–1990	450	30.4	28.0	30.1	28.3	30.8	28.3
1991–1997	818	55.2	57.5	34.5	57.3	43.3	57.3
Race/Ethnicity**							
White, non-Hispanic	1,125	75.9	65.3	55.0	65.1	52.4	65.1
Black, non-Hispanic	210	14.2	21.7	13.0	10.6	23.2	15.0
Hispanic	105	7.1	9.5	20.5	16.0	16.0	13.3
Other, non-Hispanic	42	2.8	3.4	11.4	8.4	8.4	6.7
**Region**							
Arizona	438	29.6	25.4	NA^††^	NA^††^	26.6	25.3
Arkansas	572	38.6	46.8	NA^††^	NA^††^	16.0	47.1
Georgia	472	31.9	27.8	NA^††^	NA^††^	57.4	27.7
**Respondent type**							
Self-report	1,174	79.2	77.3	99.4	99.4	100.0	100.0
Proxy report	288	19.4	21.3	0.6	0.6	0.0	0.0
Missing	20	1.4	1.4	0.0	0.0	0.0	0.0
**Severity**							
Severe	494	33.3	28.7	NA^§§^	NA^§§^	NA^§§^	NA^§§^
Nonsevere	988	66.7	71.3	NA^§§^	NA^§§^	NA^§§^	NA^§§^

**Sources:** Congenital Heart Survey To Recognize Outcomes, Needs, and well-beinG (CH STRONG, 2016–2019); National Health and Nutrition Examination Survey (NHANES, 2015–2018); Behavioral Risk Factor Surveillance System (BRFSS, 2016–2018).

**Abbreviation:** NA = not applicable.

* Standardized to CH STRONG eligible population (n = 9,312) by sex, birth cohort, maternal race/ethnicity, place of birth, and CHD severity.

^†^ NHANES 2015–2018 data used as general population comparison group, standardized to CH STRONG eligible population by sex, birth cohort, and race/ethnicity. The NHANES population is based on nationally representative sample of noninstitutionalized, civilian U.S. population.

^§^ BRFSS, 2016–2018 data from Arizona, Arkansas, and Georgia used as general population comparison group, standardized to CH STRONG eligible population by sex, birth cohort, race/ethnicity, and current residence.

^¶^ Approximate birth year was calculated for the general population by subtracting survey year of completion by survey age. For NHANES, the survey cycle midpoint was chosen to calculate birth cohort. For BRFSS, reported year of survey was used to calculate birth cohort.

** Maternal race/ethnicity was used to standardize the CH STRONG respondents to the CH STRONG eligible population. NHANES and BRFSS self-reported race/ethnicity was used to standardize to the CH STRONG eligible population.

^††^ Data by region applicable only to CH STRONG and BRFSS.

^§§^ Data by congenital heart defect severity applicable only to CH STRONG.

Compared with the general population, adults with CHDs were more likely to report a history of congestive heart failure (4.3% versus 0.2%, p<0.001) and stroke (1.4% versus 0.3%, p<0.001), but there were no significant differences for hypertension or myocardial infarction (Figure). Adults with severe CHDs were significantly more likely than the general population to have had at least one cardiac comorbidity (19.8% versus 9.0%, p<0.05), but those with nonsevere CHD (11.0%) were not.

**FIGURE:**
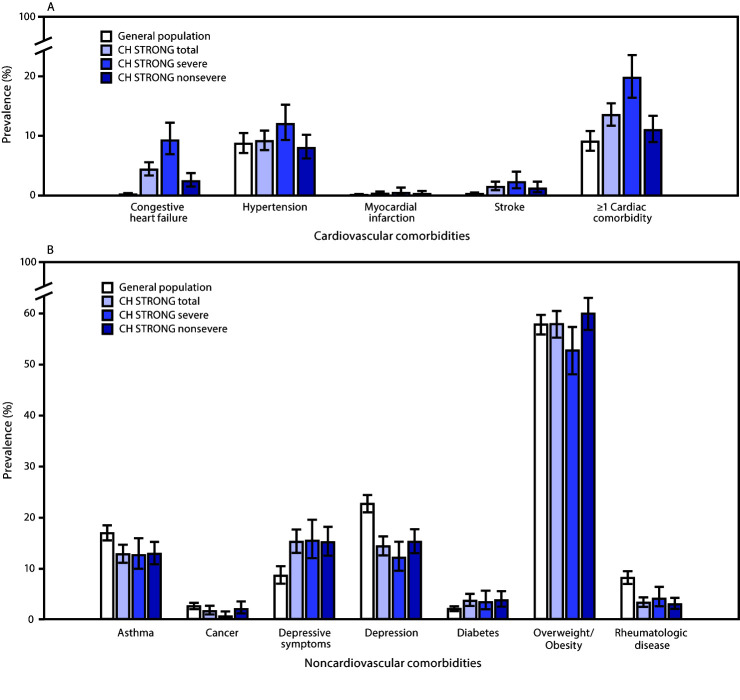
Prevalence of cardiovascular* (A) and noncardiovascular^†^ (B) comorbidities among adults aged 20–38 years with congenital heart defects^§^ (CHDs) compared with the general population aged 20–38 years — United States,^¶^ 2015–2019 **Sources:** Congenital Heart Survey To Recognize Outcomes, Needs, and well-beinG (CH STRONG, 2016–2019); National Health and Nutrition Examination Survey (NHANES, 2015–2018); Behavioral Risk Factor Surveillance System (BRFSS, 2016–2018). * General population group for history of diagnosis for congestive heart failure, hypertension, myocardial infarction, stroke, and ≥1 cardiac comorbidity is from NHANES, 2015-2018, standardized to CH STRONG eligible population by sex, age group, and race/ethnicity. ^†^ General population group for history of diagnosis for asthma, cancer, depression, diabetes, rheumatologic disease, and overweight/obesity is from the state-based Arizona, Arkansas, and Georgia BRFSS, 2016-2018 data used as general population comparison group, standardized to CH STRONG eligible population by sex, age group, and race/ethnicity. For depressive symptoms, the general population is from NHANES, 2015-2018, standardized to CH STRONG eligible population by sex, age group, and race/ethnicity; the CH STRONG population excluded responses by proxy report (CH STRONG denominator = 1,174). Participants with a score of ≥3 on the Patient Health Questionnaire-2 were considered to have depressive symptoms at time of survey completion. Overweight/obesity defined as body mass index ≥25 kg/m^2^ based on self-reported height and weight. ^§^ Full list of congenital heart defect lesions included in CH STRONG has been previously published (https://doi.org/10.1016/j.ahj.2019.12.021). Common severe lesions included single ventricle lesions, endocardial cushion defects, tetralogy of Fallot, transposition of the great arteries, truncus arteriosus, coarctation of the aorta, and interrupted aortic arch. ^¶^Compared with the general population, the total CH STRONG population was more likely to report history of congestive heart failure, stroke, and current depressive symptoms and were less likely to report history of asthma, depression diagnosis, or rheumatologic disease. Those with severe CHDs reported a lower prevalence of cancer and overweight/obesity compared with the general population, and those with nonsevere CHDs reported a higher prevalence of diabetes compared with the general population.

Persons with CHDs were more likely to report depressive symptoms at the time of the survey than those in the general population (15.1% versus 8.5%, p<0.001), but were less likely to have reported a prior diagnosis of depression (14.2% versus 22.6%, p<0.001). Adults with CHDs were also less likely than the general population to have asthma (12.7% versus 16.9%, p<0.001) or rheumatologic disease (3.2% versus 8.0%, p<0.001) (Figure).

Results differed by severity of CHDs for cancer, diabetes, and overweight/obesity when compared with the general population. Those with severe CHDs reported a lower prevalence of cancer (0.5% versus 2.5%, p<0.001) and overweight/obesity (52.7% versus 57.8%, p = 0.048) compared with the general population, but no difference for diabetes. Those with nonsevere CHDs reported a higher prevalence of diabetes (3.7% versus 2.0%, p = 0.03) compared with the general population, but no differences for cancer or overweight/obesity.

Within CH STRONG, cardiovascular comorbidities were more common among persons with severe CHDs than among those with nonsevere CHDs: history of congestive heart failure (9.3% versus 2.1%, aOR = 4.4), hypertension (12.2% versus 7.1%, aOR = 1.9), and stroke (2.4% versus 0.8%, aOR = 3.8). Overall, persons with severe CHDs had 2.4 times the odds of reporting one or more cardiac comorbidity. The prevalence of noncardiovascular comorbidities was similar in the two groups (Table 2).

**TABLE 2:** Prevalence and adjusted odd ratios of comorbidity history among adults aged 20–38 years with congenital heart defects (CHDs), by CHD severity — Congenital Heart Survey To Recognize Outcomes, Needs, and well-beinG (CH STRONG), 2016–2019

Comorbidity	Severe*		Nonsevere		Severe versus nonsevere
	Total (n = 494)	Unstandardized, % (95% CI)	Total (n = 988)	Unstandardized, % (95% CI)	aOR^†^ (95% CI)
**Cardiovascular comorbidities**					
Congestive heart failure	46	9.3 (7.1–12.2)	21	2.1 (1.4–3.2)	4.4 (2.5–7.5)
Hypertension	60	12.2 (9.6–15.3)	70	7.1 (5.6–8.9)	1.9 (1.3–2.8)
Myocardial infarction	3	0.6 (0.2–1.9)	3	0.3 (0.1–0.9)	1.5 (0.3–8.1)
Stroke	12	2.4 (1.4–4.2)	8	0.8 (0.4–1.6)	3.8 (1.5–9.7)
≥1 Cardiac comorbidity^§^	97	19.6 (16.4–23.4)	96	9.7 (8.0–11.7)	2.4 (1.7–3.3)
**Noncardiovascular comorbidities**					
Asthma	60	12.2 (9.6–15.3)	130	13.2 (11.2–15.4)	0.9 (0.6–1.2)
Cancer	3	0.6 (0.2–1.9)	16	1.6 (1.0–2.6)	0.4 (0.1–1.3)
Current depressive symptoms^¶^	56	14.6 (11.4–18.4)	114	14.7 (12.3–17.3)	1.0 (0.7–1.5)
Depression	63	12.8 (10.1–16.0)	154	15.6 (13.5–18.0)	0.9 (0.6–1.2)
Diabetes	12	2.4 (1.4–4.2)	28	2.8 (2.0–4.1)	0.8 (0.4–1.7)
Overweight/Obesity**	250	50.6 (46.2–55.0)	573	58.0 (54.9–61.0)	0.8 (0.6–1.0)
Rheumatologic disease^††^	17	3.4 (2.2–5.5)	31	3.1 (2.2–4.4)	1.2 (0.7–2.3)

**Abbreviations:** aOR = adjusted odd ratio, CI = confidence interval.

* Common severe lesions included single ventricle lesions, endocardial cushion defects, tetralogy of Fallot, transposition of the great arteries, truncus arteriosus, coarctation of the aorta, and interrupted aortic arch.

^†^ Unstandardized CH STRONG estimates adjusted for sex, maternal race/ethnicity, birth cohort, and site.

^§^ Composite variable of adults with ≥1 cardiovascular comorbidities.

^¶^ Participants with a score of ≥3 on the two-item Patient Health Questionnaire-2, with proxy report removed. In CH STRONG, 1,174 self-report participants answered both questions.

** Overweight/obesity defined as body mass index ≥25 kg/m^2^ based on self-reported height and weight.

^††^ Arthritis, gout, lupus, or fibromyalgia.

## Discussion

Advancements in the medical and surgical treatment of CHDs have led to a growing population of adults living with these conditions (*6*). With this growth has come an increasing need to understand the medical needs of these persons, especially as they age. These CH STRONG findings indicate that adults with CHDs are more likely than are adults in the general population to experience significant cardiovascular comorbidities, such as congestive heart failure or stroke. Regarding noncardiovascular comorbidities, results varied when comparing adults with CHDs with the general population. Persons with severe CHDs were more likely to have cardiovascular comorbidities than were those with nonsevere CHDs; the odds of having noncardiovascular comorbidities was similar between the two groups.

Similar to these findings, a recent study of a German nationwide registry of patients with CHDs found comorbidities to be common, with 57% of patients with CHDs aged <40 years having at least one comorbidity, including 22% with diseases of the circulatory system (*7*). Consistent with the increased odds of current depressive symptoms among CH STRONG participants, a recent international collaborative study noted that adults cared for at a CHDs center, especially those with cyanotic heart disease, were more likely than were those in the general population to report depressive symptoms (*8*). In contrast to the findings of CH STRONG, an analysis of U.S. commercial claims data during 2010–2016 among privately insured adults aged 18–40 years found higher rates of not only cardiovascular comorbidities but also noncardiovascular comorbidities among those with CHDs compared with a matched cohort without CHDs (*9*). In the same study, persons with severe CHDs had lower risks of cardiovascular comorbidities than did those with nonsevere CHDs, but higher risks of noncardiovascular comorbidities. The differences in findings between CH STRONG and the study of U.S. commercial claims data might be explained by methods used to identify CHD patients, inclusion of only those with recent medical encounters, differences in age distribution, timeframe for comorbidities, or varying definitions of CHD severity.

The findings in this report are subject to at least four limitations. First, patient-reported outcomes can be limited by low health literacy or inaccurate recall. This limitation might affect the overall prevalence estimates among the CH STRONG and general populations; however, given that questions on the CH STRONG and general population surveys were identical, the comparisons between groups are expected to be valid. Second, if those with CHDs were more likely to suffer mortality from certain comorbidities (e.g., cancer) than the general population, the results might be subject to survivor bias. Third, the data from CH STRONG were from AZ, AR, and GA, whereas the available comparison group for cardiovascular comorbidities was from the national NHANES sample. These groups may not be directly comparable, despite attempts to standardize the NHANES data to the CH STRONG population by various demographics. Finally, these findings might be limited by the CH STRONG response rate of 24%. The standardization methods previously described were employed to minimize the effects of potential response bias.

Based on these CH STRONG findings, adults with CHD might be more likely to experience cardiovascular morbidity, particularly those with severe CHDs. CHD severity does not appear to be associated with the prevalence of certain noncardiovascular comorbidities. These findings can inform providers, policy makers, patients, and families of the expectations and needs of a growing population of adults with CHDs. Awareness and education efforts aimed at clinicians can help improve the care across the lifespan for this population. Public health practitioners and clinicians can encourage young adults with CHDs to seek appropriate medical care to help them live as healthy a life as possible.

**Summary**

**What is already known about this topic?**

There are now more adults than children living in the United States with congenital heart defects (CHDs), but their long-term outcomes are unknown.

**What is added by this report?**

In the 2016–2019 Congenital Heart Survey To Recognize Outcomes, Needs, and well-beinG, young adults with CHDs were more likely than young adults in the general population to report significant cardiovascular comorbidities such as congestive heart failure or stroke. Prevalence of noncardiovascular comorbidities did not differ by congenital heart defect severity.

**What are the implications for public health practice?**

Public health practitioners and clinicians can encourage young adults with CHDs to seek appropriate medical care to help them live as healthy a life as possible.
